# Fungal Community Structure and As-Resistant Fungi in a Decommissioned Gold Mine Site

**DOI:** 10.3389/fmicb.2017.02202

**Published:** 2017-11-09

**Authors:** Silvia Crognale, Alessandro D'Annibale, Lorena Pesciaroli, Silvia R. Stazi, Maurizio Petruccioli

**Affiliations:** Department for Innovation in Biological Systems, Food and Forestry, University of Tuscia, Viterbo, Italy

**Keywords:** fungal community, high throughput sequencing, gold mine, nuclear ribosomal internal transcribed spacers profiling, arsenic resistant fungi, arsenic volatilization

## Abstract

Although large quantities of heavy metal laden wastes are released in an uncontrolled manner by gold mining activities with ensuing contamination of the surrounding areas, there is scant information on the mycobiota of gold-mine sites. Thus, the present study was aimed to describe the fungal community structure in three differently As- and Hg-polluted soils collected from the Pestarena decommissioned site by using Illumina® metabarcoding. Fungal richness was found to increase as the contamination level increased while biodiversity was not related to the concentrations of inorganic toxicants. Within the phylum Zygomigota which, irrespective of the contamination level, was predominant in all the soils under study, the most abundant genera were *Mucor* and *Mortierella*. The relative abundances of Basidiomycota, instead, tended to raise as the contamination increased; within this phylum the most abundant genera were *Cryptococcus* and *Pseudotomentella*. The abundance of Ascomycota, ranging from about 8 to 21%, was not related to the contamination level. The relative abundances of those genera (i.e., *Penicillium, Trichoderma*, and *Chaetomium*), the cultivable isolates of which exhibited significant As-resistance, were lower than the set threshold (0.5%). Mass balances obtained from As-exposure experiments with these isolates showed that the main mechanisms involved in counteracting the toxicant were accumulation and, above all, volatilization, the respective extents of which ranged from 0.6 to 5.9% and from 6.4 to 31.2% in dependence of the isolate.

## Introduction

Gold mines are major sources of environmental contamination (Ngole-Jeme and Fantke, 2017) and the contribution of auriferous mining to elevated concentrations of toxic elements heavy metals and metalloids in the environment is widely reported and documented in several countries (Ferreira da Silva et al., 2004; Fashola et al., 2016). Transition metal(loid)s accompanying gold in ores, such as, arsenic, copper, zinc, and iron in minerals, such as, pyrite and pyrrhotite, preferentially bind with cyanide in the gold extraction process (Donato et al., 2007). The high release of metal(loid)s in gold mining activities make their environmental and ecological impact highly severe since a complex of stresses are exerted on higher plants and microorganisms of the surrounding environment.

Considering that microorganisms play an essential role in biogeochemical cycling and affect both the speciation and bioavailability of metal(loid)s, it is essential to obtain a more comprehensive knowledge of the taxonomic and functional diversity of the microbial communities in metal(loid)-contaminated sites. In this respect, a thorough investigation of fungal communities in these extreme habitats is of paramount importance since fungi represent a significant portion of the biodiversity and biomass in soils and they play key roles in the maintenance of the ecosystem functioning. The use of high throughput next generation sequencing (NGS) has led to a dramatic increase in the resolution and detectable spectrum of diverse fungal phylotypes from a variety of ecosystems including natural environments (Baldrian et al., 2016; Mundra et al., 2016; Schimann et al., 2017) and heavy metal- and PAH-contaminated sites (Bourceret et al., 2016; Narendrula-Kotha and Nkongolo, 2017).

However, to the best of our knowledge, these powerful techniques have not used to characterize the mycobiota of gold mine sites yet with the only exception of a study which focused the attention on arbuscular mycorrhizal fungi (Sun et al., 2016). To fill this gap of information, one of the main objectives of this study was to investigate the fungal community in a decommissioned gold mine site using the NGS method relying on the Illumina metabarcoding. To this aim, the Pestarena mine site where gold extraction and processing activities dates back to the late 1600s (Giuliani et al., 2015) was used as the site of concern. In this area, the strong association of gold with As-containing iron sulfides, such as, for instance, arsenopyrite, had led to a historical contamination with this metalloid in addition to mercury, which had been used to facilitate the recovery of gold from its ores in the past centuries. Arsenic, as all other metal(loid)s, often impose a long-term or permanent selective pressure on species thus resulting in the development of either metal(loid)-tolerance and/or metal(loid) resistance (Turpeinen et al., 2004; Sheik et al., 2012). Moreover, chronically polluted sites are valuable isolation sources of metal(loid)-adapted populations and species that may help to design appropriate remediation strategies relying on bioaugmentation (Puglisi et al., 2012). For this reason, in the present study, the attention was also focused on the cultivable fungal isolates obtained from the Pestarena site in order to assess As-induced morphological changes and possible presence of the functional trait of As-resistance.

## Materials and methods

### Soil characteristics

Soil samples were collected from the Pestarena gold mine complex located in the Anzasca Valley, in the northern Piemont region (Italy). The history of the mining activity at that site was documented throughout the Middle Ages up to the year 1961, when the mine and the ore processing plant were decommissioned (Preite et al., 2007). Mine tailings and wastes, associated with large amounts of arsenopyrite minerals, have often been disposed in open air. Thus, the As contamination is a consequence of its release due to the oxidation of sulfide minerals contained in ore and tailing dumps. With regard to the botanical assemblage in the area, among tree species, there was the predominant presence of silver fir (*Abies alba*) with an average tree density of 500 trees ha^−1^; moreover, few specimens of other species, such as, spruce (*Picea abies*) and beech (*Fagus sylvatica*) were also found. The under-canopy vegetation was made of grasses, including mainly perennial ryegrass (*Lolium perenne*), Timothy Grass (*Phleum pratense*), and Orchard grass (*Dactylis glomerata*). The plot areas were identified so as to ensure a botanical composition as homogeneous as possible to mitigate the differences due to the plant effect and to have a horizontal distance of each sampling point larger than 3 m from the trunk of possibly present silver fir trees. Three sampling plots (50 × 50 m) were identified, two of which inside the mineral ores processing plant (45°57′28″N, 8°0′53″E and 45°57′29″N, 8°0′56.5″E) and the third one located 300 m apart from the perimeter of the plant (45°57′36.5″N, 8°1.1′56″E). In particular, for each plot area, after removal of the turf, five soil cores (~500 g), were collected at 20 cm-depth, four of which taken at the vertices of the plot and the fifth at the crossing of the diagonals. The five cores were subjected to coning and quartering to yield two samples (around 600 g) from each plot. From each soil sample, one subsample (300 g) was air-dried and then sieved (<2.0 mm) and destined to chemical analysis and fungal isolation and another subsample (50 g) was frozen (−20°C) for extraction of nucleic acids. As shown in Table 1, the soils thus obtained exhibited a sandy loamy texture and, besides to TOC, they differed for their contents in Pb, Hg and, above all, As. Since the most prominent difference among the samples was due to their As contents, for ease of identification, they will be referred to as high As, medium As and low As soils from here onwards. Regardless of the soil, the As and Hg contents were well above the maximum permissible limits in agricultural soils (20 and 1.0 mg Kg^−1^) set out by the No 1881/2006 EC Directive.

**Table 1:** Main properties of the three soils from the Pestarena gold mine site.

**Parameter**	**Soil**		
	**Low As**	**Medium As**	**High As**
Sand	77	80	79
Silt	16	10	11
Clay	7	10	10
pH	6.2	5.5	5.3
Total organic C (%)	4.6 ± 0.2	3.0 ± 0.2	1.9 ± 0.1
Cationic Exchange Capacity (cmol kg^−1^)	20.7 ± 0.2	15.6 ± 0.1	25.4 ± 0.1
Total As (mg kg^−1^)	70.8 ± 6.8	191.0 ± 3.6	1, 042.7 ± 40.1
Water-soluble As (mg kg^−1^)	1.3 ± 0.1	13.3 ± 0.4	9.2 ± 0.2
Bioavailable As (mg kg^−1^)	3.9 ± 0.4	29.6 ± 1.0	34.7 ± 1.1
Hg (mg kg^−1^)	3.3 ± 0.7	1.7 ± 0.3	93.3 ± 0.2
Pb (mg kg^−1^)	20.5 ± 1.8	11.1 ± 0.2	38.9 ± 0.4
Cr (mg kg^−1^)	10.0 ± 1.0	1.1 ± 0.2	4.3 ± 0.9
Ni (mg kg^−1^)	6.8 ± 1.0	3.2 ± 0.3	3.8 ± 0.7
V (mg kg^−1^)	6.0 ± 0.0	3.2 ± 0.1	5.4 ± 0.9
Fe (mg kg^−1^)	6, 335 ± 81.6	7, 306 ± 2, 077	10, 504 ± 421.2
18S rDNA × 10^8^ (copy number g^−1^)	2.86 ± 0.8	2.60 ± 0.73	2.93 ± 0.17

### Soil DNA extraction

Whole genomic DNA from each soil (500 mg) was extracted in quadruplicate using the Power Soil DNA extraction Kit (Mo Bio Laboratories Inc., Carlsbad, CA) and the extracts thus obtained were pooled prior to PCR amplification. Extracted DNA, analyzed by agarose gel (1% w/v) electrophoresis followed by staining with ethidium bromide, was photographed under UV trans-illumination with a GelDoc XR digital camera (Bio-Rad, USA). Each sample was then quantified with the Qubit® 1.0 fluorometer (Life Technologies) using the Qubit® dsDNA BR assay kit (Invitrogen TM) according to the manufacturer's instructions.

### Fungal quantification

The 18S rRNA genes were used as target sequences for amplification of fungal abundance. These sequences were amplified with the primer pair FR1F (5′-AICCATTCAATCGGTAIT-3′) and FF 390R (5′-CGATAACGAACGAGACCT-3′) (Chemidlin Prévost-Bouré et al., 2011). Real-time PCR assays were carried out on LightCycler® 480 System (Roche Applied Science, USA). The quantification was based on the SYBR-green fluorescent dye, which bound to double stranded DNA during PCR amplification. Each reaction was performed in a 20 μl volume containing 5 μl of template DNA, 1 μl of each primer, and 10 μl of SYBR Green PCR Master mix (Roche, Milan-Italy). The conditions used for fungal amplification were modified by increasing primer concentration (0.400 vs. 0.250 μM) and slightly increasing annealing temperature (53 vs. 50°C). Melting curve analysis of the PCR products was conducted following each assay to confirm that the fluorescence signals were due to specific PCR products and not to other artifacts, such as, for instance, primer-dimer formation.

Fungal concentrations were calculated based on a standard curve obtained using triplicate 10-fold serial dilutions (10^2^ − 10^10^ 18S copy number) of known concentration of plasmid pGem T-easy cloning vector containing the target gene as the insert. The amplified 18S rDNA PCR product was purified using PCR solution purification kit (Machery-Nagel, Germany), ligated into pGemT-easy cloning vector (Promega, Madison, WI) and cloned into *Escherichia coli* DH5α. Clones containing correct inserts were chosen as the standards for qPCR. Plasmid DNA was isolated using plasmid extraction kit (Sigma, Saint Louis, MO). As the size of the vector and PCR insert were known, the copies of 18S rRNA gene were directly calculated from the concentration of extracted plasmid DNA.

### Fungal metagenomic analysis

Sequencing was done on the Illumina MiSeq R platform (Caporaso et al., 2012) at the Igabiotech (Udine, Italy). The NEXTflex™ 18S ITS Amplicon-Seq Kit (Bioo Scientific Corporation, Austin TX) was used to prepare multiplexed amplicon libraries that span both the ITS1 and ITS2 hypervariable Internal Transcribed Spacer (ITS) regions of eukaryotic 18S ribosomal fungal rRNA genes. Quime pipeline was used for chimera filtering, grouping of replicate sequences, and to sort sequences according to decreasing abundance. Quality ITS reads were aligned and clustered into OTUs (97% similarity) and consensus sequences were generated by using Uclust version 3.0, and assigned against UNITE database (Kõljalg et al., 2013) by using the Basic Local Alignment search Tool algorithm Blastn version 2.2.2. Good's coverage index was calculated to assess the coverage reached at the selected rarefaction level.

### Fungal isolation

Autochthonous fungi were isolated from a suspension obtained by adding 9 ml sterile physiological solution to 1 g soil and magnetically stirring the solution for 30 min. For the isolation of fungi, aliquots of the suspension as such or serially diluted (10–10^3^) were spread onto Petri plates containing Rose Bengal agar (RBA) (HiMedia, Mumbai, India) supplemented with chloramphenicol (0.01%, w/v). Plates were then incubated at 28°C for 7 d and different fungal morphotypes were isolated on the basis of their phenotypic characteristics, such as, mycelium and colony morphology, spores color and the possible presence of pigments (Figure S3). Each single colony, picked with a sterile loop, was isolated again from the same medium. The pure cultures were maintained in RBA slants at 4°C and transferred every month.

### Taxonomic identification of cultivable as-resistant fungi

Genomic DNA (gDNA) of fungal isolates was extracted using Ultra Clean Microbial DNA Isolation Kit (Mobio, Qiagen, Carlsbad CA). Amplification of the ITS region of rRNA gene was performed using ITS1 and ITS4 (White et al., 1990). The optimal amplification conditions were previously reported (Crognale et al., 2012). The purified PCR products were used in sequencing reactions with the same set of primers, using a BigDye Terminator cycle sequencing ready reaction kit, version 3.0 (Applied Biosystems, Foster City, CA). Sequencing was performed on an ABI 3730XL DNA sequencer (Applied Biosystems). Sequences were compared with those of all known fungal species available in the database Gen-Bank (http://www.ncbi.nlm.nih.gov) and then deposited in the same database.

### Arsenic resistance assays in solid and liquid media

All the pure fungal isolates were first tested for their resistance to pentavalent As by growing them on agar media in the presence of variable concentrations of arsenate. In particular, the RBA medium was amended with 250, 500, 1,000, 5,000, and 10,000 mg l^−1^ of As in the form of sodium arsenate (NaH_2_AsO_4_). Plugs (Ø 8 mm) from pure cultures of each fungal isolate were inoculated individually onto 90-mm Petri plates containing RBA added with the aforementioned As concentrations. All plates were incubated at 28°C for 14 d and diametric growth (mm) measured on a daily basis during the incubation period. Appropriate control plates on As-lacking RBA were prepared, inoculated with each of the isolates and incubated in parallel under the same conditions. Resistance Index (RI) was calculated by referring diametric growth rate (DGR) of each isolate in the presence of As to that in its absence after 7 d incubation.

The most resistant isolates were then tested for their abilities to grow on Potato Dextrose broth (PDB) added with 10 mg l^−1^ of As in the form of arsenate. Erlenmeyer flasks (500 ml) containing 100 ml PDB were inoculated with five RBA plugs (Ø 8 mm) withdrawn from the edge of 7-d-old pure fungal cultures and incubated in duplicate at 28°C under orbital shaking (130 rpm) for 7 d. Flasks were closed with rubber caps internally covered with filter paper trap, previously soaked with 10% AgNO_3_ and dried overnight at 60°C in the dark. Non inoculated controls were prepared to determine background volatilization of arsenic, while fungal controls (biotic controls) cultivated in the absence of arsenic were prepared to assess growth in the absence of metal stress. At the end of the incubation, liquid cultures were filtered under vacuum through 0.45 μm filters (Whatman, Maidstone, UK), the biomass was washed with distilled water to completely remove the culture medium and the washing solution added to the final filtrate. The biomass, quantitatively recovered from the filter, was lyophilized and then held in a desiccator under vacuum prior to its gravimetric determination. An aliquot of the biomass was then digested for As determination, as described in subsection Arsenic Quantification. The pH of culture filtrates was measured prior to the addition of wash-down water (HANNA Instruments S.r.l, Italy).

### Arsenic quantification

Filters (200 mg), biomass (50 mg), and culture filtrates (2.0 ml) were transferred separately into the digestion vessels and subjected to wet oxidation using concentrated, ultra-high purity nitric acid (7.5 mL, from Carlo Erba reagent) added with 0.5 mL of concentrated hydrochloric acid (Carlo Erba reagent) and 2 ml H_2_O_2_ 35%. Oxidation was carried out in heavy-duty vessels (HDV) using a high-pressure microwave digestion oven (Mars plus, CEM Corporation, Matthews, NC, USA). Samples were ramped to 180°C over 37 min and held at 180°C for 15 min before cooling to below 50°C. The volumes of digested samples were then adjusted to 20 ml using ultrahigh purity water and used for As determination. The amount of As in culture filtrates was determined by means of Hydride Generation with inductively coupled plasma-optical emission spectroscopy (HG-ICP-OES) using an OptimaTM 8000 DV ICP-OES (Perkin-Elmer® Corp., Norwalk, CT, USA) coupled to a FIAS Manifold Hydride Kit (Perkin-Elmer® Corp). The quantity of As in both fungal biomass and filters, instead, was measured in ICP-OES equipped with an ultrasonic nebulizer (USN Perkin-Elmer® Corp., Norwalk, CT, USA). All analytical determinations were performed in triplicate.

### Statistical analysis

Data of fungal growth on both agar and liquid media were analyzed by one-way analysis of variance followed by *post-hoc* multiple pair-wise comparisons of means by the HSD-Tukey test (*p* ≤ 0.05) using SigmaStat 3.5 software. Log-transformed data were centered and unit variance-scaled prior to Partial Least Square (PLS) regression analysis, which was performed to understand whether response variables (i.e., the frequencies of most representative fungal genera) were affected by some descriptor variables [i.e., pH, TOC, CEC, contents in total, and water-soluble As (WS-As), Hg, Pb, Fe, Cr, Ni, and V] in the three soils under study. Significant PLS components were found by cross-validation and the possible presence of moderate or strong outliers checked by the distance to model in the X/Y space (DModX and DModY) and Hotelling T^2^, respectively. To determine which among the aforementioned descriptors had the predominant impact on the most abundant taxa, the variable influence in projection was used (VIP). The VIP is a weighed sum of squares of the PLS loading weights taking into account the amount of explained Y-variation in each dimension. The rule “greater than one” was employed for identifying those descriptors with the largest influence on the projection (Lindgren et al., 1996). A permutation testing, based on response permutation plot, was applied to each modeled response variable in order to perform internal validation and to evaluate the possible occurrence of over-fitting (background correlation; Lindgren et al., 1996). Residual prediction deviation (RPD), calculated as the ratio between the standard deviation of reference data and the bias-corrected Root Mean Square Error of Prediction (RMSEP), was determined to evaluate the robustness of the model. To this aim, the Sigma 13.0 software package (Umetrics, Umea, Sweden) was used.

## Results

### Fungal community analysis of gold mine site soils

Fungal abundances in the three Pestarena soils, determined by q-PCR of 18S rRNA gene, did not significantly differ each one another in their respective gene copy number which amounted to around 10^8^ gene copy number g^−1^ soil (Table 1).

The Illumina Miseq data were processed in order to eliminate those sequences that did not meet the stringent quality control criteria. The sequences derived from the filtering process were then aligned, clustered into OTUs and used to determine their phylogenetic affiliations. The rarefaction analysis, plotting the operational taxonomic unit (OTU) richness as a function of sequencing depth (Figure S1), indicated that the libraries provided an adequate sampling of fungal diversity of the three soils under study and this was also confirmed by the high values of the Good's coverage (Table 2). Among the soils of concern, the High As had a significantly higher richness values than the Low As and Medium As as indicated by both the Chao1 index (2,499 vs. 1,382 and 835, respectively) and the number of observed OTUs (955 vs. 571 and 500, respectively; Table 2).

**Table 2:** Good's coverage and biodiversity indices of the mycobiota in the High As, Medium As, and Low As soils from the Pestarena gold mine site.

**Soil**	**Good's coverage**	**Chao1 index**	**Number of observed OTU^†^**	**Shannon-Wiener index (H')**
High As	98.8	2, 499 ± 218	955 ± 72	3.91 ± 0.18
Medium As	99.3	1, 382 ± 32	571 ± 10	2.61 ± 0.08
Low As	99.5	835 ± 29	500 ± 2	3.67 ± 0.13

† *Operational Taxonomic Unit*.

**Figure 1:**
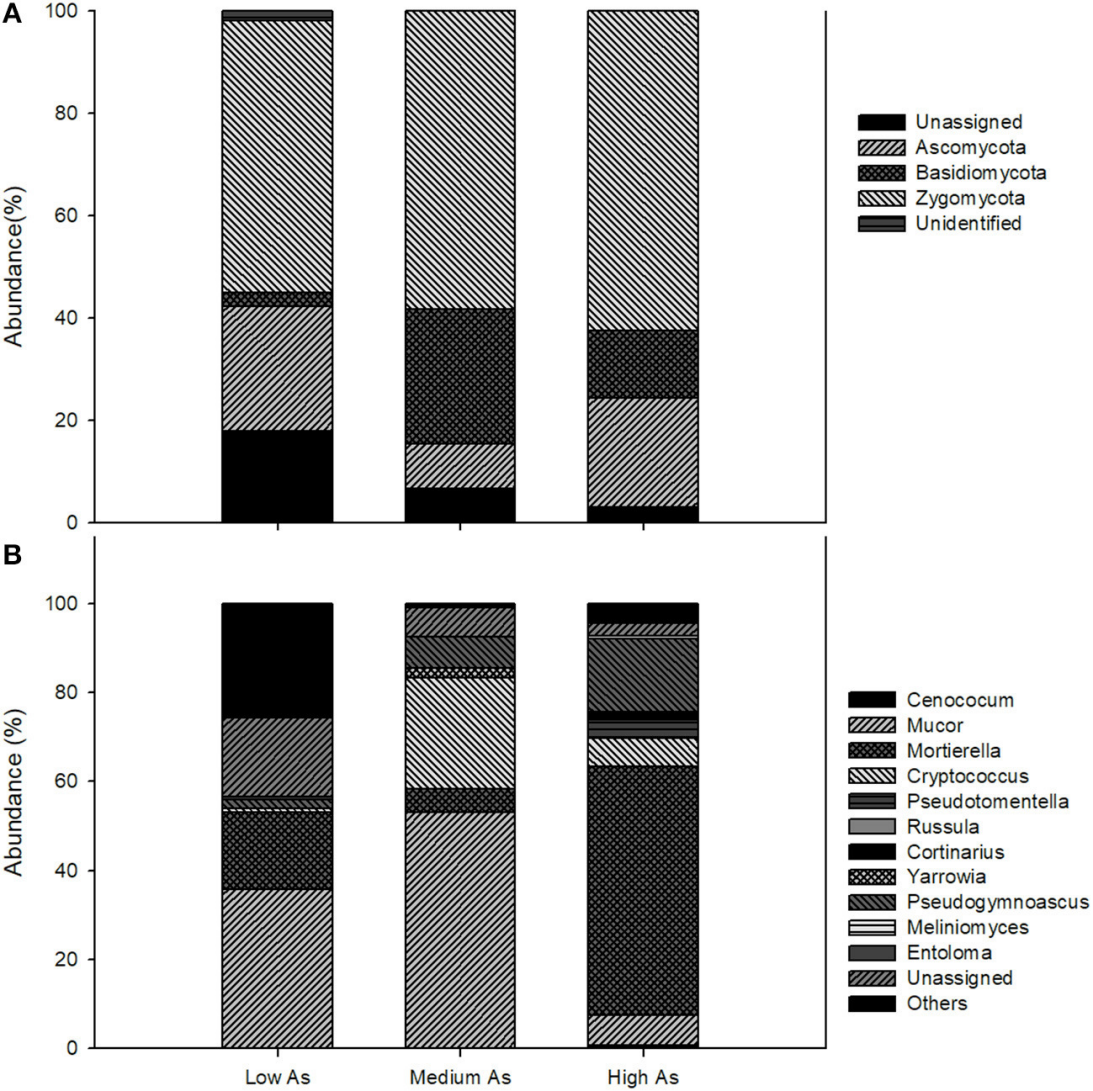
Composition of fungal communities in Pestarena soils (High As, Medium As, Low As) at the phylum **(A)** and at the genus **(B)** level. Data are the mean of two replicates (see section Soil Characteristics) using a threshold of relative sequence abundance higher than 0.5%.

At the phylum level, the fungal communities of the High As, Medium As and Low As were dominated by Zygomycota, the relative abundances of which were 62.4 ± 5.9, 58.3 ± 2.8, and 53.2 ± 10.2%, respectively (Figure 1A). Within this phylum, the most abundant orders were Mortierellales and Mucorales with *Mortierella* and *Mucor* being the largely predominant genera (Figure 1B). The relative frequencies of Ascomycota were akin in High As and Low As (21.4 ± 4.5 and 24.4 ± 6.2%, respectively), while they amounted to only 8.83 ± 2.5% in Medium As. Basidiomycota, instead, were highest and lowest in the High As and Low As soils (26.1 ± 1.9 and 2.6 ± 0.1%, respectively) and the majority of their sequences matched members of the order Tremellales and Thelephorales, the most abundant genera being *Cryptococcus* and *Pseudotomentella*, respectively. The latter genus, encompassing several ectomycorrhizal species, however, was only found in the High As soil along with other genera of ectomycorrhizal fungi, such as, *Russula* and *Cortinarius*. It is noteworthy that the saprotrophs found in the Pestarena soils were dominated by Zygomycota, which are characterized by a cell wall mainly composed of chitosan (Ruiz-Herrera, 1992), a polymer with well-known chelating properties toward metal(loid)s, including As (Da Sacco and Masotti, 2010).

**Figure 2:**
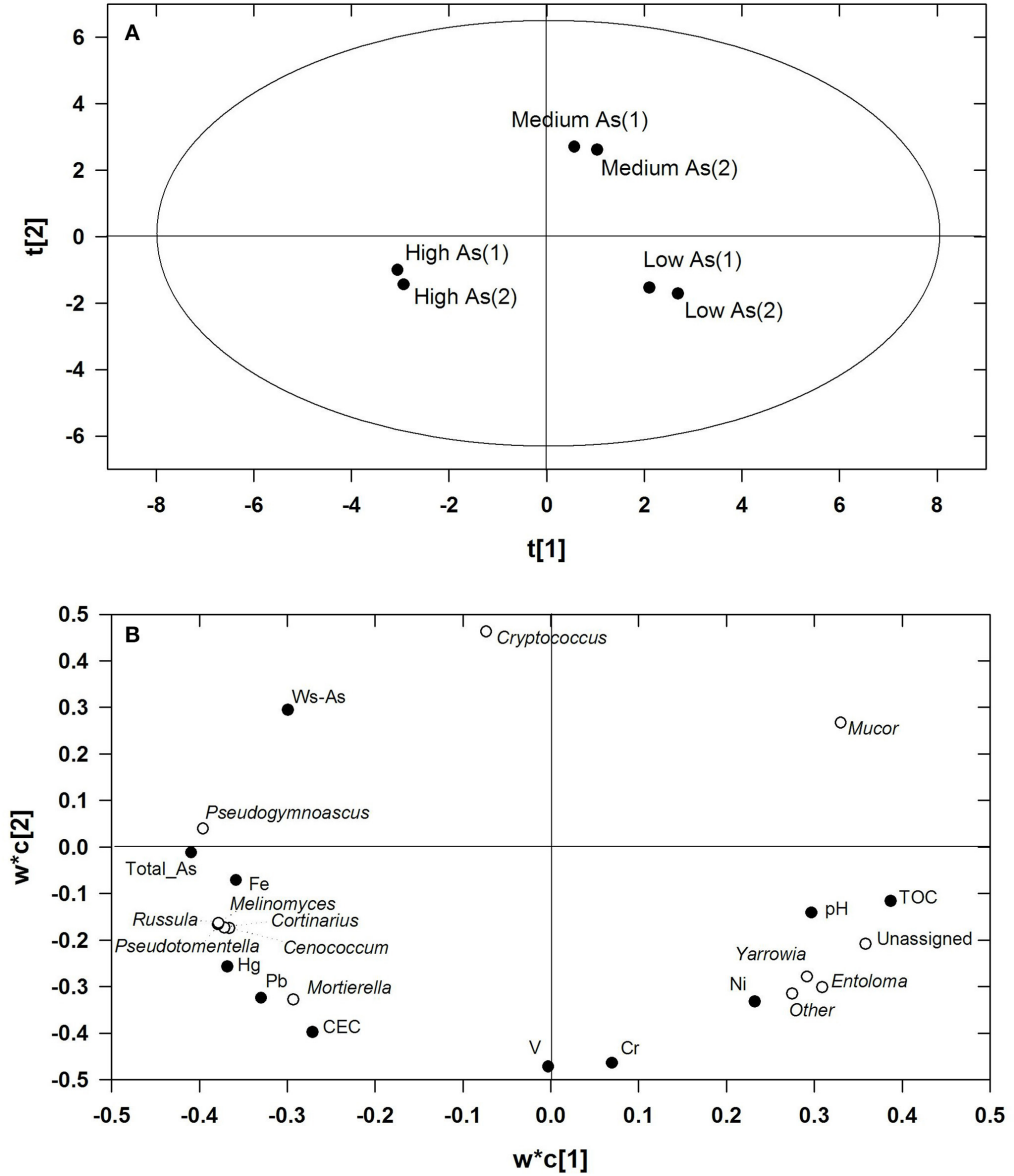
Scores **(A)** and loadings plot **(B)** of the PLS model relating the relative frequencies of most abundant fungal genera to the descriptors of the High As, Medium As, and Low As soils. The fraction of variance of all the response variable explained by the first and second latent PLS component amounted to 68.8 and 29.9%, respectively.

Figures 2A,B show the scores and the loadings plots of the PLS model which related the relative frequencies of most abundant fungal genera to the descriptors of the High As, Medium As, and Low As soils. Two components were significant, as assessed by cross-validation, the former, and the latter explaining 68.8 and 29.9% variance in response variables, respectively, and neither strong nor moderate outliers were detected as assessed by Hotelling T^2^ and DModX/DModY, respectively. The risks of over-fitting and over-prediction of the PLS model were ruled out by permutation tests, involving repeated and random reordering of the Y-block and recalculating R^2^ and Q^2^. The two regression lines fitting R^2^- and Q^2^-values of the permuted models led to Y-intercepts below 0.3–0.4 for the former parameter and below 0.05 for the latter thus meeting the validity requirements set by Lindgren et al. (1996) (Table S1). Figure 2A shows that the three soils were clearly separated along the first PLS component where the main variables driving separation were TOC, total As, Hg, and Pb, among the descriptors, and the ectomycorrhizal genera *Russula, Cortinarius, Pseudotomentella*, and *Meliniomyces*, among the response variables (Figure 2B). As opposed to *Mortierella*, the genus *Mucor*, which was most abundant in the Medium As soil, located in the diagonally opposite quadrant to that of total As, Hg and Pb indicating that its abundance was negatively correlated with these contaminants. The genera *Yarrowia* and *Entoloma* located instead in the lower right quadrant corresponding to the Low As soil in the diagonally opposite quadrant to that of WS-As. The most influential descriptors on the relative frequencies of most abundant taxa were ranked according to their respective VIP-values. In order of decreasing importance, and based on the greater than one rule, the relevant descriptors were: total As (1.137), TOC (1.108), Hg (1.091), Fe (1.077), Pb (1.060), and Ws-As (1.004). Total As and Hg exerted a marked effect on the majority of genera, the relative abundances of which were positively correlated with these parameters with the notable exception of *Mucor*. With the exceptions of the variables “Unassigned” and “Other fungi,” the RPD-values, calculated as the ratio between the standard deviation of each response variable and the respective bias-corrected RMSEP, were higher than 4.0. A range of RPD values between 2.4 and 3.0 is considered poor and the models could be applied only for very rough screening, while RPD-values >3.0 could be considered fair and recommended for screening purposes and good for quality control, respectively (Sinelli et al., 2008).

### Isolation and characterization of as-resistant fungi

In the present investigation, 14 fungal morphotypes, isolated on the basis of their shape, color colony, and spore morphology (Figure S2) were identified by sequencing the ITS (Table S2). Five strains belonged to the *Penicillium* genus, three to *Mortierella*, two to *Trichoderma*, while the others were identified as *Mucor moelleri*, and *Trichocladium asperum*. With regard to the isolates LAS_G and LAS_H, as the sequencing of the ITS region gave a percent of identity lower than 99% with *Chaetomomium* sp. and, *Tolypocladium* sp., respectively, their genus assignment was confirmed by sequencing 18S rDNA (KY773574 and KY773575, respectively). For the MAS_N and LAS_A isolates, assigned to the *Penicillium* and *Trichoderma* genera, respectively, the identification at the species level was not possible although the former showed a very high percent identity with *Penicillium onobense* (98.7%) and *Penicillium skrjabinii* (98.7%) and the latter with *Trichoderma paraviridiscens* (99.5%), *Trichoderma artroviride* (99.5%), and *Trichoderma viridescens* (99.6%).

Table 3 summarizes the results of resistance tests of the isolates of concern grown on PDA supplemented with As concentrations ranging from 250 to 10,000 mg l^−1^. Among the isolates under study, *M. moelleri* LAS_E showed the lowest tolerance to As and its RI was dramatically reduced at 1000 mg l^−1^. *Trichoderma* sp. LAS_A, *T. asperum* LAS_F and all the strains belonging to the *Mortierella* genus (LAS_B, LAS_C, and HAS_R) were able to withstand As concentration as high as 5,000 mg l^−1^ and the respective RI-values ranged from 0.11 to 0.36. *Trichoderma virens, Chaetomium* sp. and, above all, the isolates belonging to the genus *Penicillium* were the most tolerant ones due to their abilities to grow in the presence of 10,000 mg l^−1^ As. Morphological changes with respect to biotic controls were also found for some isolates exposed to high As concentration. In particular, a markedly decreased sporulation and the disappearance of the typical concentric conidiation ring were observed in As-exposed *T. virens* HAS_Q cultures as compared to control ones (Figure S4A). Exposure to As, instead, resulted in a shift from filamentous to the yeast-like form in *M. moelleri* LAS_E, as shown in Figure S4B.

**Table 3:** Resistance index and diametric growth rate (DGR) of fungal isolates grown on PDA agar plates in the absence and in the presence of increasing concentrations of sodium arsenate (250–10,000 mg L^−1^).

**Fungal isolate**	**Resistance index (RI) and diametric growth rate (DGR, mm d**^**−1**^**) in the presence of As concentrations equal to:**										
	**0**	**250**		**500**		**1,000**		**5,000**		**10,000**	
	**DGR**	**RI**	**DGR**	**RI**	**DGR**	**RI**	**DGR**	**RI**	**DGR**	**RI**	**DGR**
*Trichoderma* sp.	10.5 ± 0.7^bE^	1.0^bA^	11.0 ± 0.7^bG^	1.0^bBC^	10.7 ± 0.7^bG^	0.9^bBC^	10.0 ± 0.6^bH^	0.1^aAB^	1.1 ± 0.1^aBC^	0^aA^	0^aA^
*Mortierella alpina*	8.1 ± 0.5^dD^	0.7^bA^	6.0 ± 0.3^cCD^	0.7^bAB^	4.9 ± 0.3^bcCD^	0.5^bAB^	4.3 ± 0.2^bDEF^	0.2^aABC^	0.3 ± 0.0^aA^	0^aA^	0^aA^
*Mortierella* sp.	9.3 ± 0.6^dDE^	0.9^cA^	8.3 ± 0.5^cdEF^	0.8^cABC^	7.0 ± 0.4^cEF^	0.5^bAB^	4.9 ± 0.3^bEF^	0.2^aABC^	0.7 ± 0.0^aAB^	0^aA^	0^aA^
*Mucor moelleri*	11.0 ± 0.8^cE^	1^cA^	11.0 ± 0.8^cG^	0.4^bA^	4.1 ± 0.3^bBCD^	0.2^abA^	1.0 ± 0.1^aA^	0^aA^	0^aA^	0^aA^	0^aA^
*Trichocladium asperum*	5.3 ± 0.3^cC^	0.9^cA^	4.7 ± 0.3^cBC^	0.8^bcABC^	4.4 ± 0.3^cBCD^	0.6^bABC^	3.3 ± 0.2^bCD^	0.2^aABC^	0.1 ± 0.0^aA^	0^aA^	0^aA^
*Chaetomium* sp.	5.3 ± 0.4^cC^	1.0^bA^	5.6 ± 0.4^cBCD^	1.1^bC^	5.7 ± 0.4^cD^	1.0^bC^	5.4 ± 0.4^cF^	0.4^aBCD^	1.6 ± 0.1^bC^	0.2^aBC^	0.1 ± 0.0^aA^
*Tolypocladium* sp.	2.9 ± 0.2^dAB^	0.9^bA^	2.6 ± 0.2^cdA^	0.8^bABC^	2.1 ± 0.1^bcA^	0.6^abABC^	1.7 ± 0.1^bAB^	0.4^aBCD^	0.3 ± 0.0^aA^	0.4^aDE^	0.3 ± 0.0^aA^
*Penicillium griseopurpureum*	7.4 ± 0.6^bD^	1.0^bA^	7.4 ± 0.6^bDE^	1.0^bBC^	7.7 ± 0.7^bF^	1.0^bC^	7.1 ± 0.6^bG^	0.7^bDEF^	5.4 ± 0.5^bF^	0.3^aCD^	2.6 ± 0.2^aD^
*Penicillium janthinellum*	7.4 ± 0.6^bD^	0.8^cA^	6.4 ± 0.5^cdCE^	0.7^bcABC^	4.9 ± 0.4^bcB^	0.5^bAB^	3.7 ± 0.3^bCDE^	0.2^abABC^	1.7 ± 0.1^aC^	0.1^aAB^	1.0 ± 0.0^aB^
*Penicillium* sp.	3.8 ± 0.3^bABC^	0.9^aA^	3.4 ± 0.3^abA^	0.8^aABC^	3.1 ± 0.2^abAB^	0.9^aBC^	3.3 ± 0.2^bBCD^	1.0^aF^	3.5 ± 0.3^abE^	1.0^aF^	2.5 ± 0.1^aD^
*Penicillium canescens*	2.3 ± 0.2^bcA^	0.9^aA^	2.1 ± 0.2^bcA^	0.9^aBC^	2.1 ± 0.2^bcA^	1.0^aC^	2.6 ± 0.2^cBC^	0.8^aEF^	1.9 ± 0.1^bCD^	0.5^aE^	1.1 ± 0.0^aB^
*Penicillium soppii*	4.4 ± 0.4^cdBC^	0.8^aA^	3.7 ± 0.3^bcdAB^	0.8^aABC^	3.6 ± 0.3^bcABC^	0.8^aBC^	3.4 ± 0.3^bcCDE^	0.6^aDE^	2.7 ± 0.3^abDE^	0.5^aE^	2.3 ± 0.1^aD^
*Trichoderma virens*	11.0 ± 0.7^cE^	1^bA^	11.0 ± 0.8^cG^	1.0^bBC^	11.0 ± 0.7^cG^	1.0^bC^	10.4 ± 0.7^cH^	0.5^aCDE^	5.4 ± 0.3^bF^	0.2^aBC^	1.9 ± 0.1^aC^
*Mortierella* sp.	11.0 ± 0.8^dE^	0.9^cA^	10.0 ± 0.7^cdEFG^	0.9^cBC^	9.9 ± 0.6^cdG^	0.8^cBC^	8.4 ± 0.5^cG^	0.4^bBCD^	4.0 ± 0.3^bE^	0^aA^	0^aA^

*Multiple comparisons of data were carried out by the Tukey test: mean values with the same superscript letter were not significantly different (P ≤ 0.05). Lowercase and uppercase letters refer to comparisons among row and column means, respectively*.

Those isolates, which were able to withstand the highest As concentration in agar medium, were subsequently grown in liquid cultures on the PDB medium to assess the possible occurrence of adaptation mechanisms actuated under As disturbance, such as, bio-accumulation and/or bio-volatilization. After a preliminary setup phase, aimed at finding appropriate exposure conditions, the As concentration was set at 10 mg l^−1^ because of its compatibility with the containment of incubation times.

**Figure 3:**
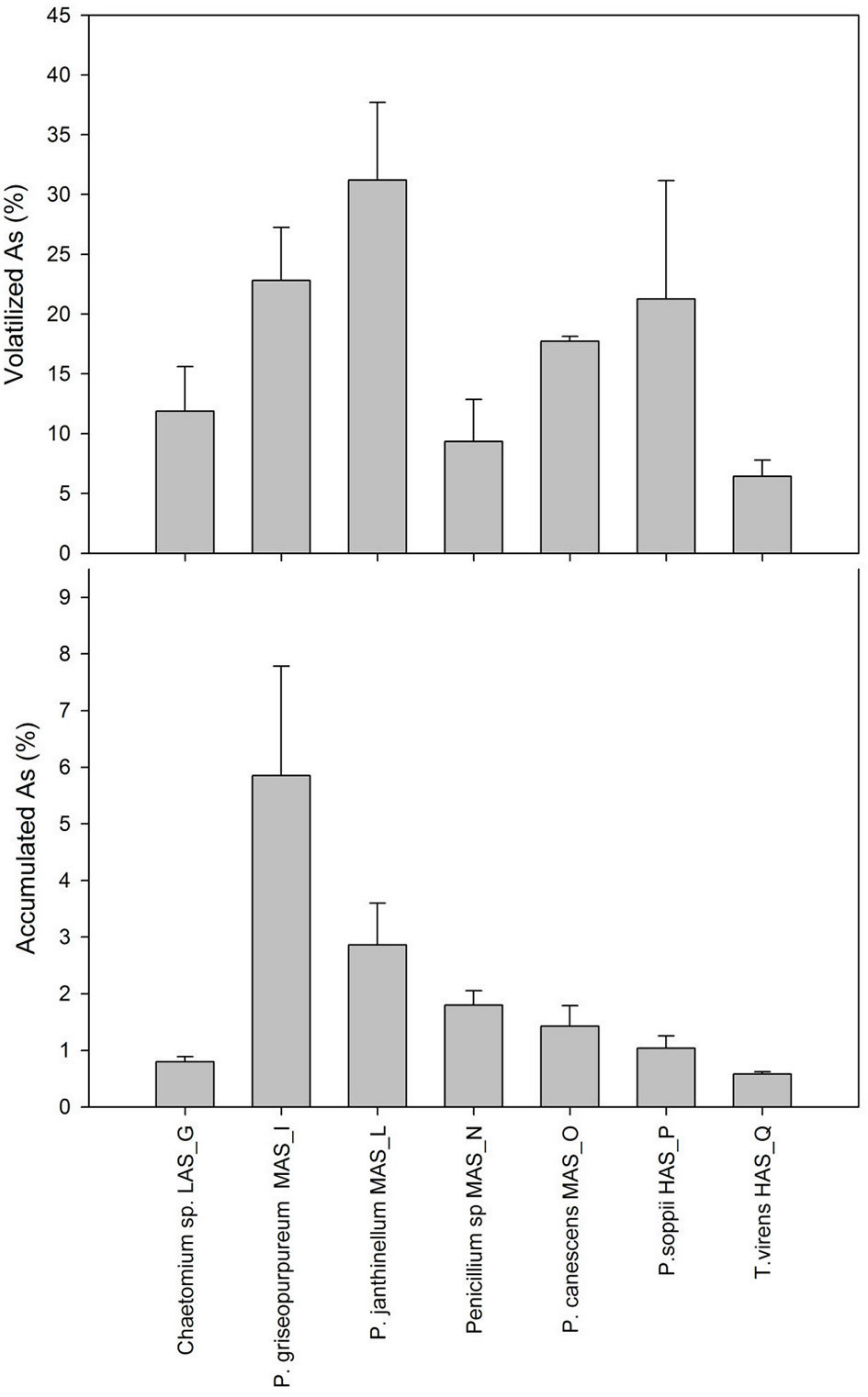
Percentage of As volatilization **(A)** and accumulation **(B)** in 7-d-old shaken cultures of the fungal strains isolated from Pestarena soils and exposed to 10 mg L^−1^ As, supplied as sodium arsenate.

All the strains under study grew actively in the presence of As, as indicated by both the high biomass productions and the high values of relative growth with respect to the biotic controls, as shown in Table S3. Residual As in culture broths, ranging from 65.2 ± 7.0 to 92 ± 1.4%, was invariably lower than that found in the abiotic control (98.9 ± 0.2%). The main mechanism by which As was removed from the liquid medium involved volatilization the highest extent of which was found for *Penicillium janthinellum* MAS_L (31.2%) and the lowest with *T. virens* HAS_Q (6.4%; Figure 3A). Significant volatilization extents were also observed with *Penicillium soppii* (21.3%) and *Penicillium griseopurpureum* (22.8%). The highest values of specific volatilization were found for *P. soppii* (0.33 mg g^−1^ dry biomass) and *P. janthinellum* (0.26 mg g^−1^ dry biomass). Thus, As removal exerted by volatilization was significantly higher than that due to accumulation, the extent of which was generally lower than 2% with the exceptions of *P. griseopurpureum* (5.9%) and *P. janthinellum* (2.9%; Figure 3B); for these strains, the respective specific accumulations were 51 and 23 μg g^−1^ dry biomass, respectively (Table S3).

## Discussion

The majority of studies focus on the short-term changes of microbial diversity due to recent contamination events, mainly simulated at the laboratory scale and relying on the spiking of unpolluted soils. However, chronic pollution could be also an important factor able to shape the soil microbial communities in addition to environmental conditions and edaphic parameters. While the short term contamination impacts negatively on microbial abundance, richness, and diversity, in aged multi-contaminated soils, the microbial community was found to adapt toward a rich diversity pattern over time (Schimel et al., 2007; Paissé et al., 2008; Bourceret et al., 2016). These findings are in agreement with the present study showing that both microbial diversity and richness in the three aged metal-contaminated soils resembled those found in unperturbed soils (Bourceret et al., 2016; Narendrula-Kotha and Nkongolo, 2017). Noteworthy, the best richness was found in the soil characterized by the highest As and Hg contents; the positive correlations between these parameters and the relative frequencies of the majority of identified genera suggest that these taxa were well adapted to the long-term contamination.

Among the ectomycorrrhizal genera, which were found mainly in the most polluted Pestarena soil, *Cortinarius*, which is widely distributed worldwide (Zotti et al., 2014), was reported to be able to accumulate As in the cap (Slekovec and Irgolic, 1996) and *Pseudogymnoascus* was isolated from the hairy roots of *Calluna vulgaris* grown in a decommissioned arsenic mine site (Sharples et al., 2000). *Meliniomyces* was found among the major species in the belowground fungal community of metal-polluted pine forests (Op De Beeck et al., 2015) even though this genus has been found to be dominant in this kind of forests irrespective of metal contamination (Koizumi and Nara, 2017).

All the three soils under study were dominated from a quantitative viewpoint by saprotrophs, with the genus *Mucor* being the most abundant in Low As and Medium As soils. Conversely, its relative abundance markedly dropped in High As soil which was, instead, dominated by *Mortierella*. Among the identified genera, *Entoloma* appeared to be rather sensitive to both As and, above all, to WS-As (Figure 2B). With this regard, several species belonging to this genus have been reported to accumulate As mainly as arsenite and arsenate; thus, their high sensitivity to the metalloid might be due to their inability to convert it into forms devoid of toxicity such as, arsenobetaine and arsenocholine (Byrne et al., 1995; Dembitsky and Rezanka, 2003). With regard to *Cryptococcus*, it is worth noting that this genus was present among the main yeasts previously isolated from Fe- and As-rich mines (Delavat et al., 2013). A species belonging to this genus, namely, *C. musci*, was isolated from another decommissioned mine site and found to exhibit the highest tolerance to As(V) as compared to other yeasts isolated from the same site (Vadkertiová et al., 2016). The ability of some *Cryptococcus* species to tolerate high metal concentrations was already reported (Russo et al., 2016) and ascribed to their high polyphosphate accumulation capacity (Andreeva et al., 2014). Noteworthy, for another species, such as, *Cryptococcus humicola*, the As-resistance was found to be due to its ability to convert chromated copper arsenate to volatile forms, such as, trimethylarsine (Bentley and Chasteen, 2002).

Screening of cultivable As-resistant fungi from sites historically contaminated by this pollutant has been suggested to provide useful candidates for remediation purposes (Puglisi et al., 2012). For this reason, several fungal isolates obtained from the Pestarena soils were tested for their As-resistance. Among them, an isolate belonging to the genus *Mucor*, found to be dominant by NGS exhibited low As-resistance and underwent significant morphological change under As exposure switching from the filamentous to the yeast-like growth mode. A similar behavior was described for *M. circinelloides* exposed to high metal concentration (Omoifo, 2006). The exposure to As induced significant modification on another Pestarena isolate, namely *Trichoderma virens* HAS_Q, which lost the ability of producing concentric conidiation rings (rhythmic conidiation), a very typical light-responsive feature of several *Trichoderma* species (Steyaert et al., 2010). In this respect, this is the first study reporting an As-induced inhibitory effect on rhythmic conidiation. In another study, As exposure was found to enhance the degree of sporulation in *Trichoderma* sp. (Srivastava et al., 2011).

Among cultivable isolates, the most represented genus was *Penicillium* and included species, such as, *P. soppii, Penicillium canescens, P. janthinellum*, and *P*. *griseopurpureum*. Although the Arsenic resistance of this genus is well documented (Valix et al., 2001), no reports are currently available regarding this matter for those species identified in the present study, with the only exception of *P. janthinellum* (Su et al., 2010, 2012). It is noteworthy that although the indigenous isolates of the genus *Penicillium* exhibited high resistance to Arsenic in both solid and liquid cultures, the relative abundances of this genus in the three soils under study were very low (<0.05%). Although these results are in line with those of Stefani et al. (2015) who found that many of the most abundant microorganisms detected by NGS were not obtained with plate-culturing techniques, it may not be ruled out that some reads belonging to the *Penicillium* genus were allocated by the pipeline either within the “others” or the “unassigned” groups. In this respect, it has been noted that the QIIME pipeline, which was used in the present study, relies on the whole processed sequences rather than on the (hyper) variable ITS sub-regions (Balint et al., 2014), the use of which enables a highly accurate identification of genera and species. Consequently, the aforementioned low abundances of *Penicillium* detected by NGS did not necessarily mean that it was not competitive in that environment. However, the efficacy of its isolates in site-specific bioaugmentation should be validated by further experiments to monitor their persistence and activity in the target soils.

Mass balances related to As exposure studies in liquid cultures of filamentous fungi are reported comparatively in Table 4. Noteworthy, the adopted exposure concentration, which was three orders of magnitude lower than the upper concentration limit in plate assays, enabled the reduction of incubation times as opposed to other exposure studies, the end-points of which were generally 30 days (Ĉerňasky et al., 2007; Urik et al., 2007; Čerňanský et al., 2009; Boriová et al., 2014). These long incubation times on liquid media are due to different conditions of mass transfer, complexation and availability of metal(loid)s as compared to solid ones. As a consequence, the minimal inhibitory concentration (MIC), defined as the lowest concentration of the metal inhibiting fungal growth, has been reported often to be much higher in solid media than in liquid ones (Al-Kadeeb, 2007; Silva et al., 2013). Despite the low number of available studies dealing with an accurate mass balance of As, it is worth mentioning that the most recurring genera overlap only partially with those found in the present study. In particular, no *Aspergillus, Fusarium, Scopulariopsis*, and *Talaromyces* isolates were obtained from the Pestarena soils from which, instead, an interesting *Chaetomium* isolate was found. Table 4 shows that the percent volatilization and accumulation in the majority of isolates ranged from 4 to 31% and from 0.1 to 11%, respectively, with the exception of a single study where these figures were largely exceeded (Ĉerňasky et al., 2007). Thus, the predominant mechanism of As removal involved volatilization as observed for the isolates obtained in this study.

**Table 4:** Comparative mass balance of As volatilization and accumulation in liquid cultures of fungal strains as a function of absolute amounts of the metalloid and exposure time.

**Microorganism**	**IA**	**Biomass**	**Incubation**	**Volatilization**			**Accumulation**			**References**
	**(mg)**	**(g)**	**(d)**	**(mg)**	**(%)**	**(mg g^−1^)**	**(mg)**	**(%)**	**(mg g^−1^)**	
*Aspergillus* sp.	2.39	0.31	5	0.733	30.7	0.42	0.003	0.1	0.01	[1]
*A. niger*	0.25	0.56	30	0.068	27.5	0.12	0.015	6.0	0.03	[2]
*A.niger*	1.0	0.46	30	0.252	25.2	0.54	0.056	5.6	0.12	[2]
*A. clavatus*	0.25	0.35	30	0.05	20.0	0.14	0.01	4.0	0.03	[3]
*A. clavatus*	1.0	0.37	30	0.221	22.1	0.59	0.057	5.7	0.15	[3]
*A. clavatus*	2.5	0.25	30	1.522	60.9	6.21	0.736	29.4	3.00	[3]
*Penicillium* sp.	2.33	0.26	5	0.889	30.7	0.42	0.003	0.1	0.01	[1]
*P. glabrum*	0.25	0.43	30	0.063	25.2	0.15	0.171	68.4	0.40	[3]
*P. glabrum*	1.0	0.50	30	0.262	26.2	0.53	0.668	66.8	1.35	[3]
*P. janthinellum*	2.5	0.29	5	0.16	6.4	0.55	0.027	1.08	0.09	[4]
*P. janthinellum*	1.0	1.19	7	0.309	31.2	0.25	0.028	2.9	0.29	Present study
*Trichoderma viride*	0.25	0.16	30	0.010	4	0.05	0.01	4.0	0.05	[3]
*Trichoderma viride*	1.0	0.29	30	0.093	9.3	0.32	0.062	6.2	0.22	[3]
*Trichoderma asperellum*	2.5	0.17	5	0.105	4.2	0.61	0.011	0.4	0.06	[4]
*Trichoderma* sp.	1.0	0.91	7	0.064	6.4	0.07	0.006	0.6	0.006	Present study
*Talaromyces wortmanni*	0.25	0.44	30	0.027	10.8	0.06	0.029	6.0	0.027	[2]
*Talaromyces wotmanni*	1.0	0.50	30	0.090	9.00	0.18	0.114	11.4	0.23	[2]
*Talaromyces flavus*	0.25	0.51	30	0.025	10	0.05	0.025	10.0	0.05	[2]
*Talaromyces flavus*	1.0	0.56	30	0.088	8.80	0.17	0.111	11.1	0.20	[2]
*Neurosartorya fisheri*	0.80	1.36	35	0.191	28.9	0.14	0.01	1.3	0.007	[5]
*Fusarium oxysporium*	2.50	0.24	5	0.190	7.6	0.79	0.013	0.5	0.05	[4]
*Scopulariopsis brevicaulis*	0.19	n.r^†^	30	0.016	8.4	n.r^†^	0.007	3.5	0.03	[6]
*Scopulariopsis brevicaulis*	1.94	n.r^†^	30	0.195	10	n.r^†^	0.04	2.0	0.42	[6]

*Both As volatilization and accumulation are reported in terms of absolute amounts, percent with respect to initial amounts (IA) or referred to g of dry biomass*.

† *n.r., not reported; [1] (Majumder et al., 2011); [2] (Ĉerňasky et al., 2007); [3] (Urik et al., 2007); [4] (Su et al., 2010); [5] (Čerňanský et al., 2009); [6] (Boriová et al., 2014)*.

## Author contributions

SC participated in the conception of the work, in the analysis of results, and in the writing the draft of the manuscript, AD contributed to design the experiment, analysis of the data, and contributed in manuscript preparation and its critical revision, LP contributed in the execution of part of the experimental work and in the acquisition of the data, SS contributed to the chemical analysis of samples, MP coordinated the project and supervised both the study and the manuscript preparation. All authors reviewed the results and approved the final version of the manuscript.

### Conflict of interest statement

The authors declare that the research was conducted in the absence of any commercial or financial relationships that could be construed as a potential conflict of interest.

## Supplementary material

Figure S1 Satellite view, density tree, altitude, and annual meteorological data of the sampling site. Gold mine processing area is evidenced by white dashed line.

Figure S2 Rarefaction curves of observed OTUs within the 16S rRNA sequences derived from Pestarena Low As (dotted line), Medium As (dashed line), High As (solid line) soils.

Figure S3 Fungal morphotypes isolated from Pestarena soils.

Figure S4 Morphological changes in **(A)** *T. virens* HAS_Q and **(B)** *M. moelleri* LAS_E grown on Rose Bengal Agar medium added with 1,000 and 250 mg L^−1^ of pentavalent As, respectively, in the form of sodium arsenate.

## References

[B1] Al-Kadeeb A. S. (2007). Effect of lead and copper on the growth of heavy metal resistance fungi isolated from second industrial city in Riyadh, Saudi Arabia. J. Appl. Sci. 7, 1019–1024. 10.3923/jas.2007.1019.1024

[B2] Andreeva N. Ryazanova L. Dmitriev V. Kulakovskaya T. Kulaev I. (2014). Cytoplasmic inorganic polyphosphate participates in the heavy metal tolerance of *Cryptococcus humicola*. Folia Microbiol. 59, 381–389. 10.1007/s12223-014-0310-x 24531869

[B3] Baldrian P. Zrustová P. Tláskal V. Davidová A. Merhautová V. Vrška T. (2016). Fungi associated with decomposing deadwood in a natural beech-dominated forest. Fungal Ecol. 23, 109–122. 10.1016/j.funeco.2016.07.001

[B4] Bálint M. Schmidt P. A. Sharma R. Thines M. Schmitt I. (2014). An Illumina metabarcoding pipeline for fungi. Ecol. Evol. 4, 2642–2653. 10.1002/ece3.1107 25077016 PMC4113289

[B5] Bentley R. Chasteen T. G. (2002). Microbial methylation of metalloids: arsenic, antimony, and bismuth. Microbiol. Mol. Biol. Rev. 2, 250–271. 10.1128/MMBR.66.2.250-271.2002 PMC120786 12040126

[B6] Boriová K. Cernanský S. Matúš P. Bujdoš M. Šimonovičová A. (2014). Bioaccumulation and biovolatilization of various elements using filamentous fungus *Scopulariopsis brevicaulis*. Lett. Appl. Microbiol. 59, 217–223. 10.1111/lam.12266 24712346

[B7] Bourceret A. Cébron A. Tisserant E. Poupin P. Bauda P. Beguiristain T. (2016). The bacterial and fungal diversity of an aged PAH- and heavy metal-contaminated soil is affected by plant cover and edaphic parameters. Microb. Ecol. 73, 711–724. 10.1007/s00248-015-0682-8 26440298

[B8] Byrne A. R. Šlejkovec Z. Stijve T. Fay L. Goessler W. Gailer J. (1995). Arsenobetaine and other arsenic species in mushrooms. Appl. Organomet. Chem. 4, 305–313. 10.1002/aoc.590090403

[B9] Caporaso J. G. Lauber C. L. Walters W. A. Berg-Lyons D. Huntley J. Fierer N. (2012). Ultra-high-throughput microbial community analysis on the Illumina HiSeq and MiSeq platforms. ISME J. 8, 1621–1624. 10.1038/ismej.2012.8 PMC3400413 22402401

[B10] Ĉerňasky S. Urík M. J. Ševc J. Khun M. (2007). Biosorption and biovolatilization of arsenic by heat-resistant fungi. Environ. Sci. Pollut. Res. 14, 31–35. 10.1065/espr2006 21959538

[B11] Čerňanský S. Kolenčík M. Ševc J. Urík M. Hiller E. (2009). Fungal volatilization of trivalent and pentavalent arsenic under laboratory conditions. Bioresour. Technol. 100, 1037–1040. 10.1016/j.biortech.2008.07.030 18774290

[B12] Chemidlin Prévost-Bouré N. Christen R. Dequiedt S. Mougel C. Lelièvre M. Jolivet C. (2011). Validation and application of a PCR primer set to quantify fungal communities in the soil environment by real-time quantitative PCR. PLoS ONE 9:e24166 10.1371/journal.pone.0024166 PMC3169588 21931659

[B13] Crognale S. Pesciaroli L. Petruccioli M. D'Annibale A. (2012). Phenoloxidase-producing halotolerant fungi from olive brine wastewater. Process Biochem. 47, 1433–1437. 10.1016/j.procbio.2012.05.014

[B14] Da Sacco L. Masotti A. (2010). Chitin and chitosan as multipurpose natural polymers for groundwater arsenic removal and As2 O_3_ delivery in tumor therapy. Mar. Drugs 8, 1518–1525. 10.3390/md8051518 20559486 PMC2885078

[B15] Delavat F. Lett M. C. Lièvremont D. (2013). Yeast and bacterial diversity along a transect in an acidic, As-Fe rich environment revealed by cultural approaches. Sci. Total Environ. 463–464, 823–828. 10.1016/j.scitotenv.2013.06.023 23859900

[B16] Dembitsky V. M. Rezanka T. (2003). Natural occurrence of arseno compounds in plants, lichens, fungi, algal species, and microorganisms. Plant Sci. 165, 1177–1192. 10.1016/j.plantsci.2003.08.007

[B17] Donato D. B. Nichols O. Possingham H. Moore M. Ricci P. F. Noller B. N. (2007). A critical review of the effects of gold cyanide-bearing tailings solutions on wildlife. Environ. Int. 33, 974–984. 10.1016/j.envint.2007.04.007 17540445

[B18] Fashola M. O. Ngole-Jeme V. M. Babalola O. O. (2016). Heavy metal pollution from gold mines: environmental effects and bacterial strategies for resistance. Int. J. Environ. Res Public Health 13, 1–20. 10.3390/ijerph13111047 27792205 PMC5129257

[B19] Ferreira da Silva E. Zhang C. Serrano Pinto L. Patinha C. Reis P. (2004). Hazard assessment on arsenic and lead in soils of Castromil gold mining area, Portugal. Appl. Geochem. 19, 887–898. 10.1016/j.apgeochem.2003.10.010

[B20] Giuliani A. Mandrone G. Rossetti P. (2015). Preliminary studies aimed at the re-opening of Pestarena gold mine (North-Western Alps, Italy). Am. J. Environ. Sci. 11, 145–155. 10.3844/ajessp.2015.145.156

[B21] Koizumi T. Nara K. (2017). Communities of putative ericoid mycorrhizal fungi isolated from alpine dwarf shrubs in Japan: effects of host identity and microhabitat. Microb. Environ. 32, 147–153 10.1264/jsme2.ME16180 28529264 PMC5478538

[B22] Kõljalg U. Nilsson R. H. Abarenkov K. Tedersoo L. Taylor A. F. Bahram M. (2013). Towards a unified paradigm for sequence-based identification of fungi. Mol. Ecol. 22, 5271–5277. 10.1111/mec.12481 24112409

[B23] Lindgren F. Hansen B. Karcher W. Sjöström M. Eriksson L. (1996). Model validation by permutation tests: applications to variable selection. J. Chemomet. 10, 521–532. 10.1002/(SICI)1099-128X(199609)10:5/6<521::AID-CEM448>3.0.CO;2-J

[B24] Majumder A. Biswas T. Kole S. C. (2011). Biovolatilization and bioaccumulation of pentavalent arsenic by fungal strains isolated from intensively cultivated soil. J. Crop Weed 7, 249–252.

[B25] Mundra S. Halvorsen R. Kauserud H. Bahram M. Tedersoo L. Elberling B.. (2016). Ectomycorrhizal and saprotrophic fungi respond differently to long-term experimentally increased snow depth in the High Arctic. Microbiol. Open 5, 856–869. 10.1002/mbo3.375 27255701 PMC5061721

[B26] Narendrula-Kotha R. Nkongolo K. K. (2017). Bacterial and fungal community structure and diversity in a mining region under long-term metal exposure revealed by metagenomics sequencing. Ecol. Genet. Genomics 2, 13–24. 10.1016/j.egg.2016.11.001

[B27] Ngole-Jeme V. M. Fantke P. (2017). Ecological and human health risks associated with abandoned gold mine tailings contaminated soil. PLoS ONE 12:e0172517. 10.1371/journal.pone.0172517 28222184 PMC5319768

[B28] Omoifo C. O. (2006). Effect of myoinositol and zinc on sporangiospore-yeast transformation of *Mucor circinelloides* Tieghe. Afr. J. Biotechnol. 5, 707–714.

[B29] Op De Beeck M. Lievens B. Busschaert P. Rineau F. Smits M. Vangronsveld J.. (2015). Impact of metal pollution on fungal diversity and community structures. Environ. Microbiol. 17, 2035–2047. 10.1111/1462-2920.12547 24947496

[B30] Paissé S. Coulon F. Go-i-Urriza M. Peperzak L. McGenity T. J. Duran R. (2008). Structure of bacteria communities along a hydrocarbon contamination gradient in a coastal sediment. FEMS Microbiol. Ecol. 66, 295–305. 10.1111/j.1574-6941.2008.00589.x 18803671

[B31] Preite D. Ciriotti M. E. Blaß G. Appiani R. (2007). Pirrotite a Pestarena. Micro (UK Report).

[B32] Puglisi E. Hamon R. Vasileiadis S. Coppolecchia D. Trevisan M. (2012). Adaptation of soil microorganisms to trace element contamination: a review of mechanisms, methodologies, and consequences for risk assessment and remediation. Crit. Rev. Environ. Sci. Technol. 42, 2435–2470. 10.1080/10643389.2011.592735

[B33] Ruiz-Herrera J. (1992). Fungal Cell Wall: Structure, Synthesis, and Assembly. Boca Roton, FL: CRC Press.

[B34] Russo G. Libkind D. Giraudo M. R. Delgado O. D. (2016). Heavy metal capture by autochthonous yeasts from a volcanic influenced environment of Patagonia. J. Basic Microbiol. 56, 1203–1211. 10.1002/jobm.201600048 27427287

[B35] Schimann H. Bach C. Lengelle J. Louisanna E. Barantal S. Murat C.. (2017). Diversity and structure of fungal communities in neotropical rainforest soils: the effect of host recurrence. Microb. Ecol. 73, 310–320. 10.1007/s00248-016-0839-0 27645139

[B36] Schimel J. Balser T. C. Wallenstein M. (2007). Microbial stress response physiology and its implications for ecosystem function. Ecology 88, 1386–1394. 10.1890/06-0219 17601131

[B37] Sharples J. M. Chambers S. M. Meharg A. A. Cairney J. W. G. (2000). Genetic diversity of root-associated fungal endophytes from *Calluna vulgaris* at contrasting field sites. New Phytol. 148, 153–162. 10.1046/j.1469-8137.2000.00734.x 33863033

[B38] Sheik C. S. Mitchell T. W. Rizvi F. Z. Rehman Y. Faisal M. Hasnain S.. (2012). Exposure of soil microbial communities to chromium and arsenic alters their diversity and structure. PLoS ONE 7:e40059. 10.1371/journal.pone.0040059 22768219 PMC3386950

[B39] Silva R. F. Lupantini M. Trinitade L. Antoniolli Z. I. Steffen R. B. Andreazza R. (2013). Copper resistance of different ectomycorrhizal fungi such as *Pisolithus microcarpus, Pisolithus* sp., *Scleroderma* sp. and *Suillus* sp. Braz. J. Microbiol. 44, 613–621. 10.1590/S1517-83822013005000039 24294261 PMC3833167

[B40] Sinelli N. Spinardi A. Di Egidio V. Mignani I. Casiraghi E. (2008). Evaluation of quality and nutraceutical content of blueberries (*Vaccinium corymbosum* L.) by near and mid-infrared spectroscopy. Postharvest Biol. Technol. 50, 31–36. 10.1016/j.postharvbio.2008.03.013

[B41] Slekovec M. Irgolic K. J. (1996). Uptake of arsenic by mushrooms from soil. Chem. Spec. Bioavail. 8, 67–73. 10.1080/09542299.1996.11083271

[B42] Srivastava P. K. Vaish A. Dwivedi S. Chakrabarty D. Singh N. Tripathi R. D. (2011). Biological removal of arsenic pollution by soil fungi. Sci. Total Environ. 409, 2430–2442. 10.1016/j.scitotenv.2011.03.002 21459413

[B43] Stefani F. O. Bell T. H. Marchand C. de la Providencia I. E. El Yassimi A. St-Arnaud M.. (2015). Culture-dependent and -independent methods capture different microbial community fractions in hydrocarbon-contaminated soils. PLoS ONE 10:e128272. 10.1371/journal.pone.0128272 26053848 PMC4460130

[B44] Steyaert J. M. Richard J. W. Mendoza-Mendoza A. Stewart A. (2010). Reproduction without sex: conidiation in the filamentous fungus Trichoderma. Microbiology 156, 2887–2900. 10.1099/mic.0.041715-0 20688823

[B45] Sun Y. Zhang X. Wu Z. Hu Y. Wu S. Chen B. (2016). The molecular diversity of arbuscular mycorrhizal fungi in the arsenic mining impacted sites in Hunan Province of China. J. Environ. Sci. 39, 110–118. 10.1016/j.jes.2015.10.005 26899650

[B46] Su S. M. Zeng X. B. Li L. F. Duan R. Bai L. Y. Li A. G.. (2012). Arsenate reduction and methylation in the cells of *Trichoderma asperellum* SM-12F1*, Penicillium janthinellum* SM-12F4, and *Fusarium oxysporum* CZ-8F1 investigated with X-ray absorption near edge structure. J. Hazard. Mater. 243, 364–367. 10.1016/j.jhazmat.2012.09.061 23122191

[B47] Su S. Zeng X. Bai L. Jiang X. Li L. (2010). Bioaccumulation and biovolatilisation of pentavalent arsenic by *Penicillin janthinellum, Fusarium oxysporum* and *Trichoderma asperellum* under laboratory conditions. Curr. Microbiol. 61, 261–266. 10.1007/s00284-010-9605-6 20155358

[B48] Turpeinen R. Kairesalo T. Häggblom M. M. (2004). Microbial community structure and activity in arsenic-, chromium- and copper-contaminated soils. FEMS Microbiol. Ecol. 47, 39–50. 10.1016/S0168-6496(03)00232-0 19712345

[B49] Urik M. Cernanský S. Ševc J. Šimonovičová A. Littera P. (2007). Biovolatilization of Arsenic by different fungal strains. Water Air Soil Pollut. 186, 337–342. 10.1007/s11270-007-9489-7

[B50] Vadkertiová R. Molnárová J. Lux A. Vaculík M. Lišková D. (2016). Yeasts associated with an abandoned mining area in Pernek and their tolerance to different chemical elements. Folia Microbiol. 61, 199–207. 10.1007/s12223-015-0424-9 26358066

[B51] Valix M. Tang J. Y. Malik R. (2001). Heavy metal tolerance of fungi. Miner. Eng. 14, 499–505. 10.1016/S0892-6875(01)00037-1

[B52] White T. J. Bruns T. D. Lee S. B. Taylor J. W. (1990). Amplification and direct sequencing of fungal ribosomal RNA Genes for phylogenetics, in PCR - Protocols and Applications–A Laboratory Manual, eds Innis M. A. Gelfand D. H. Sninsky J. J. White T. J. (Orlando, FL: Academic Press), 315–322.

[B53] Zotti M. Ambrosio E. Di Piazza S. Bidaud A. Boccardo F. Pavarino M. (2014). Ecology and diversity of Cortinarius species (Agaricales, Basidiomycota) associated with *Quercus ilex* L. in the Mediterranean area of Liguria (North-western Italy). Plant Biosyst. 148, 357–366. 10.1080/11263504.2013.877538

