# Supplementary figures and images for: Fungal Community Structure and As-Resistant Fungi in a Decommissioned Gold Mine Site
Source: Front Microbiol. 2017 Nov 9;8:2202. doi: 10.3389/fmicb.2017.02202 (PMC5684174; doi:10.3389/fmicb.2017.02202)

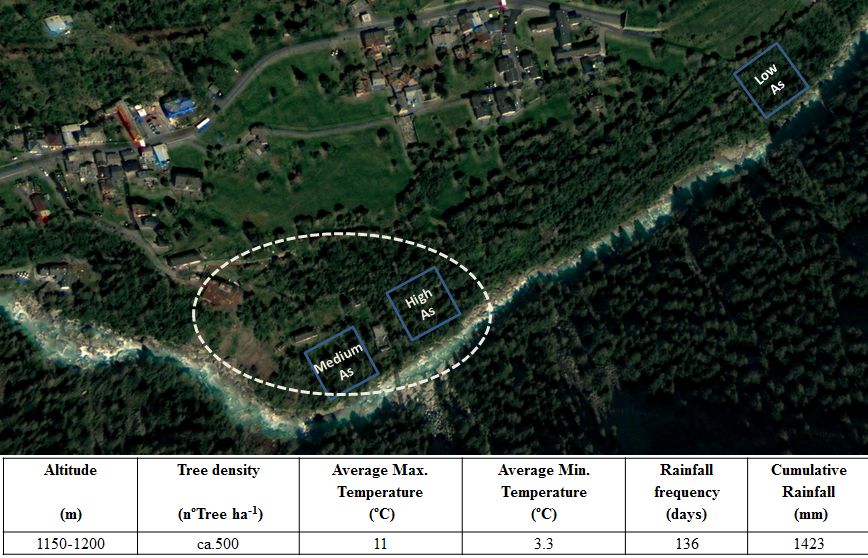

Supplement: Figure S1 — Satellite view, density tree, altitude, and annual meteorological data of the sampling site. Gold mine processing area is evidenced by white dashed line. [file Image1.JPEG]

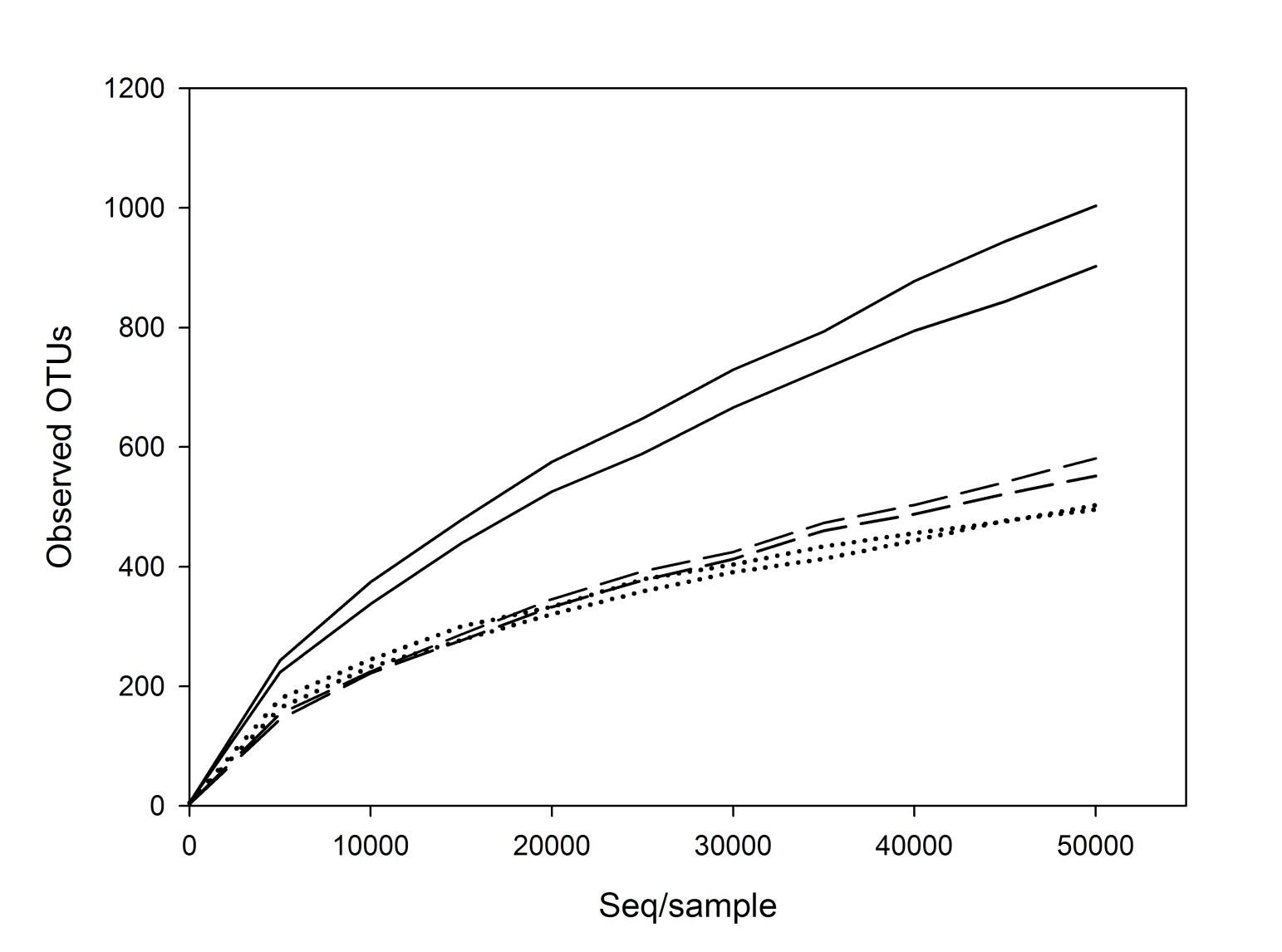

Supplement: Figure S2 — Rarefaction curves of observed OTUs within the 16S rRNA sequences derived from Pestarena Low As (dotted line), Medium As (dashed line), High As (solid line) soils. [file Image2.JPEG]

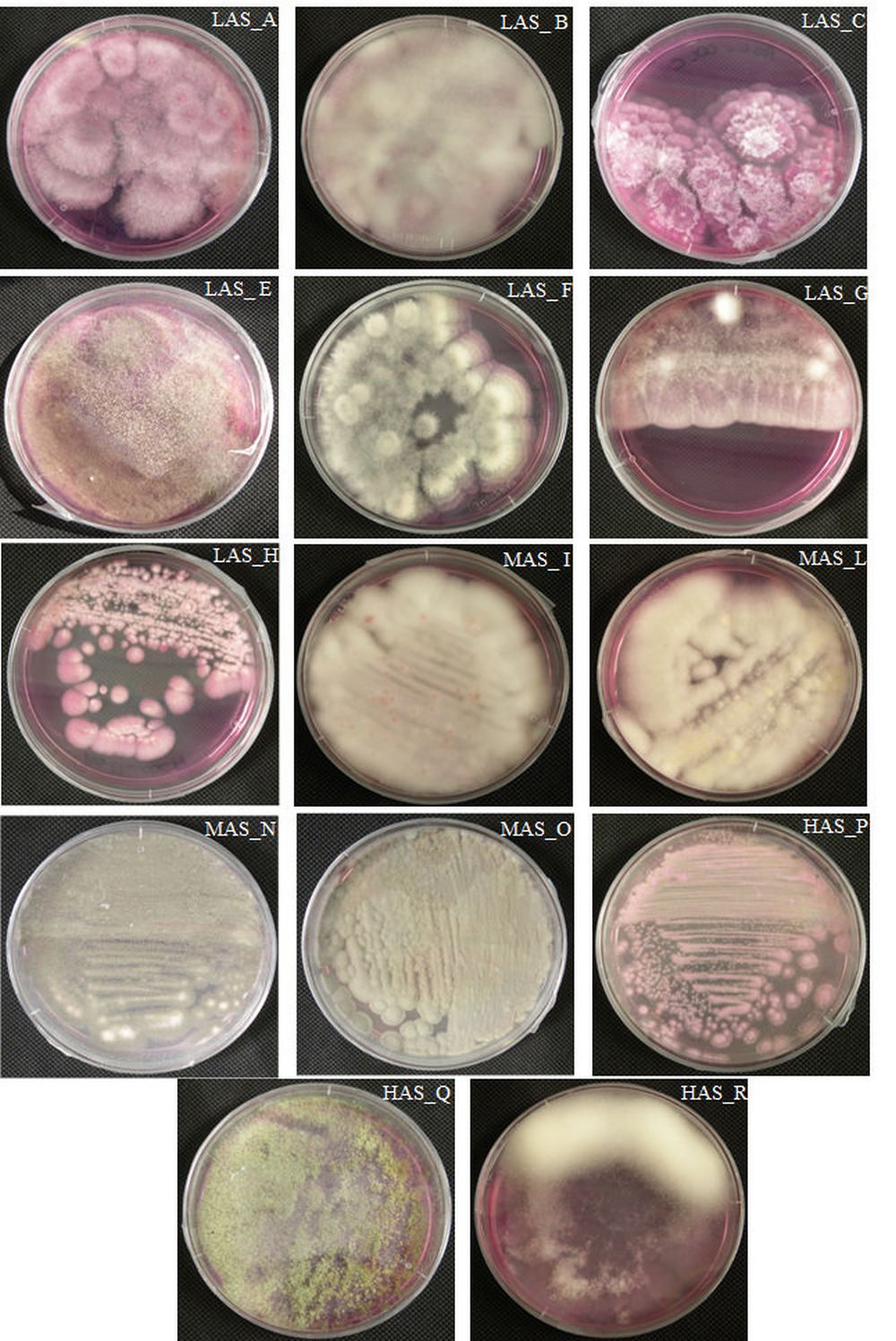

Supplement: Figure S3 — Fungal morphotypes isolated from Pestarena soils. [file Image3.JPEG]

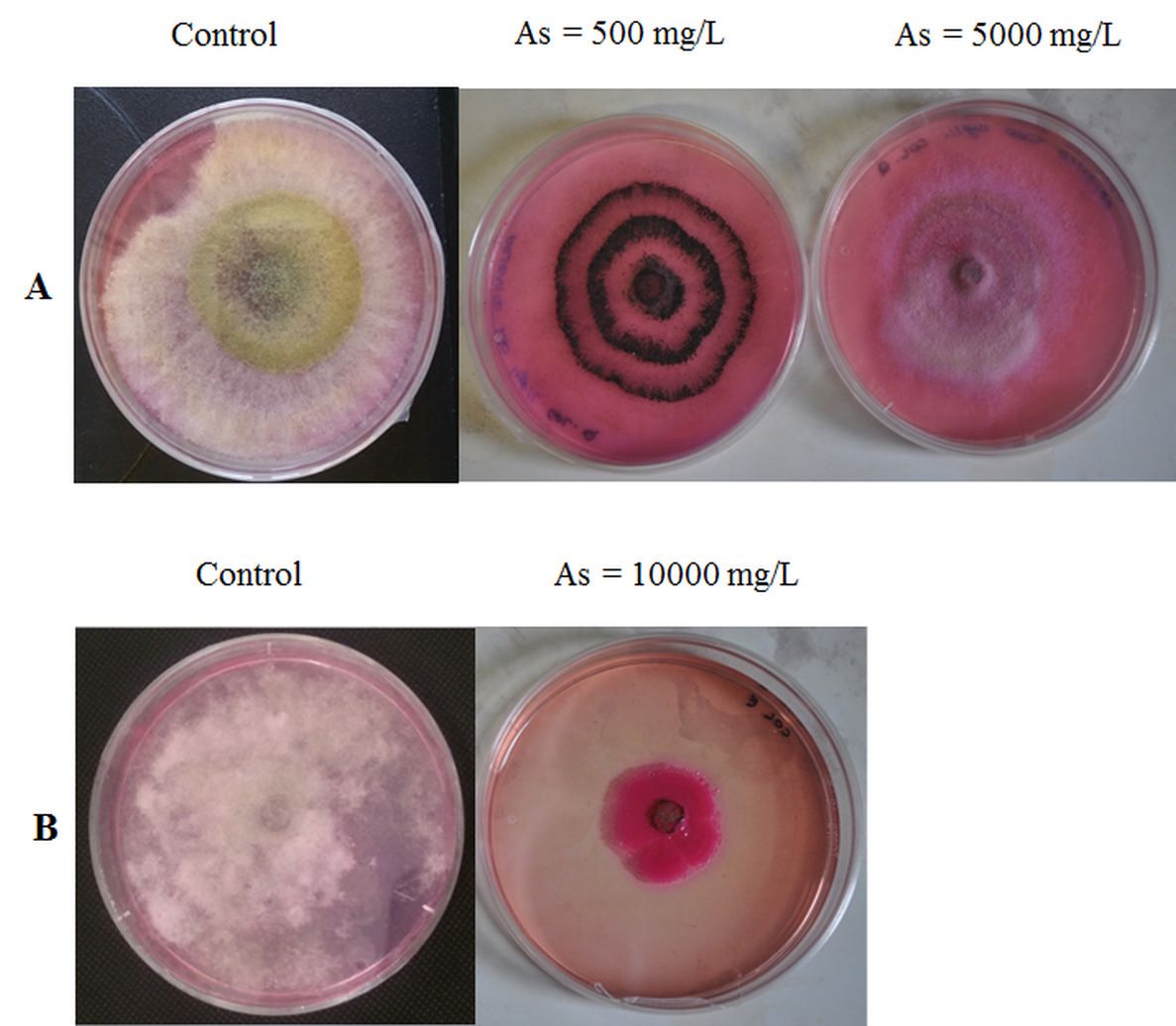

Supplement: Figure S4 — Morphological changes in (A) T. virens HAS_Q and (B) M. moelleri LAS_E grown on Rose Bengal Agar medium added with 1,000 and 250 mg L−1 of pentavalent As, respectively, in the form of sodium arsenate. [file Image4.JPEG]
