# Supplementary material for: Fungal Community Structure and As-Resistant Fungi in a Decommissioned Gold Mine Site
Source: Front Microbiol. 2017 Nov 9;8:2202. doi: 10.3389/fmicb.2017.02202 (PMC5684174; doi:10.3389/fmicb.2017.02202)
Supplement: Supplementary file 5 [file Table1.PDF]

Table S1. Model parameters of response variables including R^2, cross-validated R^2 (Q^2), Root Mean Square Error of the Estimate (RMSEE), Root Mean Square of the Prediction (RMSEP) and Residual Prediction Deviation (RPD).

Genus	R^2	Q^2	RMSEE	RMSEP	RPD
Unassigned	0.983*	0.969*	0.009	0.024	2.921
Fungi (other)	0.983 *	0.974*	0.015	0.073	1.620
Cenococcum	0.959 *	0.913*	0.001	0.002	11.406
Pseudogymnoascus	0.968*	0.899*	0.015	0.030	5.514
Yarrowia	0.971*	0.958*	0.002	0.010	5.318
Mielomyces	0.985*	0.959*	0.000	0.001	19.843
Cortinarius	0.973*	0.936*	0.001	0.002	14.049
Entoloma	0.986*	0.978*	0.000	0.001	13.477
Russula	0.987*	0.961*	0.000	0.000	25.994
Pseudotomentella	0.982*	0.954*	0.003	0.005	16.197
Cryptococcus	0.977*	0.980*	0.013	0.032	7.096
Mortierella	0.988*	0.982*	0.031	0.068	4.112
Mucor	0.964*	0.944*	0.051	0.070	4.014

(*) The asterisk denotes that the two regression lines fitting R^2 and Q^2 values of the permuted models (20 permutations) met the validity requirements set by Lindgren et al. (1996), namely Y-intercepts below 0.3-0.4 for the former parameter and below 0.05 for the latter.
