# Supplementary material for: Fungal Community Structure and As-Resistant Fungi in a Decommissioned Gold Mine Site
Source: Front Microbiol. 2017 Nov 9;8:2202. doi: 10.3389/fmicb.2017.02202 (PMC5684174; doi:10.3389/fmicb.2017.02202)
Supplement: Supplementary file 6 [file Table2.PDF]

Table S2. Taxonomic identification of fungal isolates from High As, Medium As and Low As soils of the Pestarena mine site by sequencing internal transcribed spacer (ITS) region, accession numbers of the deposited sequences and respective percent identities with those available from the NCBI GenBank database.

**Isolate**	**Identification**	**ITS size (bp)**	**Identity (%)**	**Accession number**
LAS_A	*Trichoderma* sp.	551	99.7	KY678778
LAS_B	*Mortierella alpina*	636	99.4	KY678779
LAS_C	*Mortierella* sp.	561	99.6	KY678772
LAS_E	*Mucor moelleri*	580	99.6	KY678773
LAS_F	*Trichocladium asperum*	404	100	KY678774
LAS_G*	*Chaetomium* sp.	527	98.0	KY688070
LAS_H*	*Tolypocladium* sp.	489	96.0	KY688071
MAS_I	*Penicillium griseopurpureum*	491	99.6	KY678777
MAS_L	*Penicillium janthinellum*	545	99.6	KY678780
MAS_N	*Penicillium* sp.	536	98.7	KY688069
MAS_O	*Penicillium canescens*	520	100	KY678775
HAS_P	*Penicillium soppii*	523	99.4	KY688068
HAS_Q	*Trichoderma virens*	539	99.8	KY678776
HAS_R	*Mortierella* sp.	438	98.6	KY688072

* Due to the low percent identity, the genus assignment of these isolates was also confirmed by sequencing the 18S rDNA using the Primer pair NSI and NS2 (White et al., 1990.)
