# Supplementary material for: Fungal Community Structure and As-Resistant Fungi in a Decommissioned Gold Mine Site
Source: Front Microbiol. 2017 Nov 9;8:2202. doi: 10.3389/fmicb.2017.02202 (PMC5684174; doi:10.3389/fmicb.2017.02202)
Supplement: Supplementary file 7 [file Table3.PDF]

Table S3. Fungal biomass production, pH of culture broths and mass balance of arsenic in 7-d-old shaken cultures of the fungal strains under study exposed to 10 mg L^{-1} As, supplied as sodium arsenate

Fungal isolate	pH	Biomass§ (g)	Residual As (mg)	Volatilized As (mg)	Specific volatilization (mg g^{-1})	Accumulated As (mg)	Specific As accumulation (mg)
Chaetomomium sp.	4.2±0.1	0.81±0.01 (2.6)	0.864±0.037	0.118±0.045	0.145±0.058	0.008 ± 0.001	0.010±0.001
Penicillium griseopurpureum	3.0±0.1	1.12±0.08 (1.0)	0.705±0.026	0.226±0.031	0.201±0.043	0.058 ± 0.013	0.051±0.013
Penicillium janthinellum	7.0±0.3	1.19±0.06 (1.1)	0.652±0.070	0.309±0.07	0.258±0.073	0.028 ± 0.005	0.023±0.004
Penicillium sp.	6.3±0.1	1.17±0.01 (1.0)	0.879±0.038	0.092±0.045	0.078±0.039	0.018 ± 0.002	0.015±0.002
Penicillium canescens	7.3±0.1	1.24±0.01 (1.2)	0.799±0.001	0.175±0.000	0.141±0.001	0.014 ± 0.003	0.012±0.003
Penicillium soppii	5.0±.01	0.68±0.03 (1.7)	0.767±0.097	0.211±0.095	0.330±0.052	0.010 ± 0.003	0.016±0.003
Trichoderma virens	5.3±0.5	0.91±0.06 (1.1)	0.920±0.014	0.064±0.000	0.070±0.005	0.006 ± 0.000	0.006±0.000

§ Data between round brackets are the ratios between biomass obtained in exposed and control cultures
